# Fluid balance versus weighing: A comparison in ICU patients: A single center observational study

**DOI:** 10.1371/journal.pone.0299474

**Published:** 2024-04-26

**Authors:** R. S. M. Mensink, W. Paans, M. H. Renes, W. Dieperink, F. Blokzijl

**Affiliations:** 1 Department of Intensive Care, University Medical Center Groningen, Groningen, The Netherlands; 2 Research Group Nursing Diagnostics, Hanze University of Applied Sciences, Groningen, The Netherlands; Health Services Academy, PAKISTAN

## Abstract

**Background:**

The fluid balance is a critical parameter in intensive care units (ICU) as it provides information about the patient’s volume status. However, the accuracy of fluid balance measurements is often compromised due to the complexity and repetition of actions involved. Additionally, the fluid balance could be recalculated for insensible fluid loss. Weighing is an alternative method to estimate the patient’s volume status. Built-in scales in beds make patient weighing easier and less time-consuming, allowing clinicians to intervene more quickly on existing treatments.

**Aim:**

This study compares fluid balance, and body weight changes over time in ICU patients. Furthermore, it seeks to determine the degree of congruence between the fluid balance corrected for insensible fluid loss and daily body weight in ICU patients.

**Methods:**

A single-center observational study was conducted in an ICU of a university hospital. All consecutive patients admitted to a bed with an integrated weighing scale were eligible. Exclusion criteria were (1) body weight ≥254,4 kilograms; (2) oral nutrition; (3) a flush catheter or balance; (4) only a single weight measurement; (5) delta body weight change of ≥5kg in 12 hours. Weights and fluid balances were obtained every 12 hours.

**Results:**

We obtained 2282 measurements (n = 187 patients). The correlation between weight and fluid balance was weak (r = 0.274). After adjusting the fluid balance for insensible fluid loss, the correlation remained weak (r = 0,268). Bland Altman analysis revealed a wide confidence interval for both the fluid balance and corrected fluid balance versus weight.

**Conclusion and implications of key findings:**

This study shows a weak correlation between weight and fluid balance. Therefore, when monitoring the volume status in the ICU, fluid balance and weight should both be taken into account. This two-pronged approach is crucial because it provides more control over erroneous fluid balance or weighing measurements.

## Introduction

Fluid balance monitoring in critically ill patients is essential and part of the care process and practice of nurses. Daily fluid balance is calculated as the difference between fluid intake and fluid output over a 24h period and provides an indication of the patient’s volume status [1]. Based on this volume status, fluid administration is provided as a part of hemodynamic resuscitation in the ICU [2]. The main reasons for administering fluids are to maintain organ perfusion. However, critically ill patients often have increased vascular permeability resulting in significant fluid overload. This can cause severe tissue oedema, leading to disturbed oxygen and metabolite diffusion. Fluid overload can result in impaired organ function, such as renal failure, and ultimately increases mortality risk [3, 4].

The fluid balance is automatically calculated by the EHR (Electronic Health Record). Hereby, inputs such as infusion, oral fluids, nutrition and water via feeding tube are tracked. Outputs such as drain production, diuresis, vomiting, gastric retention, and defecation are manually entered into the EHR by nurses. Depending on the clinical condition, the number of variables of the fluid balance can increase to 26 items [5, 6]. The complexity, number and repetition of actions ensure that it is difficult for nurses to track in- and outputs accurately. Therefore, registration of the fluid balance is often inaccurate [5, 7].

Moreover, the insensible fluid losses (IFL) caused by sweating and breathing are not measurable and hence not accounted for in routine practice [8]. However, studies indicate fever-related fluid loss can be as much as 500 ml per day [9]. Contrarily, mechanical ventilation results in a reduction in IFL by humidification of the inhaled air, reducing the amount of water losses. Considering these two components are crucial in the ICU, the estimation of a corrected fluid balance should be considered [9].

Another commonly used non-invasive technique to estimate the patient’s daily fluid balance is weighing. Beds with built-in weighing scales make it easy to determine the patient’s daily body weight. Short-term variations in body weight are almost exclusively associated with changes in body fluids [10]. The Acute Physiology and Chronic Health Evaluation (APACHE) IV score serves as a prognostic indicator for assessing the severity of illness, consequently offering insight into the length of stay (LOS) among ICU patients [11]. Considering body weight measurements may provide a more accurate estimate of the fluid balance, further investigation in accuracy is needed [10, 12].

Several studies have been conducted in the ICU to compare fluid balance with daily weighing, but recent studies remain scarce [2]. Given that technological progress is rapid, and beds with integrated weighing scales are getting better over the years, the assumption could be that these studies no longer adequately represent the current state of practice. Schneider et al. reported that weighing ICU patients daily proved difficult, and adherence was poor. There was a weak correlation between changes in body weight and fluid balance [10]. Another study found that weighing in beds was not sufficient [13]. A more recent study showed a good correlation between changes in body weight and cumulative fluid balance, however this study only weighed every day at 7 AM [2]. It is, therefore, uncertain whether the calculation of the fluid balance is interchangeable with weighing.

Finally, it is important to gain more insight into the agreement between these methods because clinicians can intervene more quickly in existing treatments, weighing is less time-consuming and, therefore, can be conducted more often during the day.

## Aims

This study compares the measurements of fluid balance and changes in body weight over time in ICU patients. The secondary aim focuses on the extent to which the fluid balance, corrected for IFL, is congruent with the changes in the daily body weight of ICU patients.

## Method

### Design

We designed a single-center observational study in an ICU of a Dutch university hospital with 35 ICU beds. Participants were not assigned to a specific group or according to rules but received regular treatment [14]. A convenience sampling strategy was used to include as many patients as possible [14].

### Population and domain

The study population included all consecutive ICU patients admitted to a bed with weighting capability. The study was conducted from February till May 2023.

### Exclusion criteria

Patients who met one of the following criteria were excluded from the study: (1) patients taking oral food because body weight can be expected as less reliable once a patient consumes oral food [15]; (2) patients weighing more than 254.5 kilograms (kg), the upper limit the bed could measure [16]; (3) patients with a flush catheter or flush balance considering the errors in the her; (4) patients with a short admission resulting in only a single body weight measurement; (5) Isolated changes in body weight greater than 5 kg over the course of 12 hours were excluded from the analysis since they were unlikely to reflect physiological values (e.g. measurement error).

### Sample size

A power analysis was used to determine the sample size. The power has been set at 0.8, with an alpha of 0.05. The range of agreement in the Bland Altman analysis is defined as mean bias ± 2 SD [17]. A pilot study was conducted in December 2022, which yielded a mean standard difference of -0.28 and a standard deviation of 1.89 This study, therefore, required 2096 measurement pairs for a limit of agreement (Loa) of 4,19 [18].

### Data collection

To gain insight into the patient characteristics of the study population, age, gender, APACHE IV score, reason of admission and ICU length of stay (LOS) were collected.

Daily at 12:00 PM and 12:00 AM, the body weight in kg and the calculated fluid balance in milliliters were determined by the ICU nurses for all included ICU patients.

Every 12 hours, the patient’s maximum body temperature in degrees Celsius and whether they were still intubated was documented to use Cox’s formula for IFL to calculate the corrected fluid balance.

### Data analysis

Collected data were analyzed using SPSS, version 29 (IBM Corp. Armonk, NY). Patient demographics were displayed using descriptive statistics. Continuous variables were presented with a mean and standard deviation. Categorical data were displayed by using frequency and percentage.

The degree of congruence between the two measurement methods (fluid balance and body weight) was chosen because there is no gold standard which measurement in this regard can be compared to.

A histogram was created to verify the normal distribution of the collected data [10]. A Pearson correlation coefficient was run first to demonstrate the relation between fluid balance and body weight. Data pairs consisted of the difference between two consecutive fluid balance measurements and the corresponding body weight. Weight data were corrected for additional materials before analysis. Next, a Bland Altman plot was used to demonstrate comparability between fluid balance, which is the standard measurement method and the body weight [19]. Since the length of the patient’s stay may have impact on muscle degeneration, measurements from the first week of ICU admission were compared to the second week of ICU admission.

For each patient, we first compared the fluid balance (FB) with the body weight (BW). Secondly, we corrected the fluid balance for insensible fluid losses (IFL) using Cox formula [9]. The first Bland Altman plot illustrates changes in the delta fluid balance and simultaneous the delta body weight (Fig 2). The second Bland Altman plot illustrates changes in the corrected fluid balance and simultaneous the body weight (Fig 3).

### Missing data

Collected data was utilized for the overall comparison between fluid balance and body weight. In cases, the percentage of the missing data was minimal (≤5%), and there were not more than two consecutive missing measurements, the missing data was imputed by dividing the difference between the measurement after and before the missing values [20, 21].

### Study procedures

#### Fluid balance

All ICU nurses were trained to calculate a fluid balance per protocol. The her calculated inputs such as infusion, nutrition and water via feeding tube and oral fluids. Outputs such as drain production, diuresis, vomiting, stomach retention and defecation were manually documented by nurses in therEHR.

#### Study beds

We used MultiCare ‘verstelbaar intensive care bed’ ICU beds (Linet, Slany, Czech Republic) [16]. This ICU bed is certified according to European standards and quality systems. According to the manufacturer, the bed is able to accurately estimate the patient’s weight with a precision of 0.5kg [16]. We performed a small pilot study, comparing the weights of 150 healthy volunteers using a medically certified weighing chair and the MultiCare bed. The results showed a mean difference of 0.075 and confirms that the MultiCare bed is indeed weighing accurately (S1 Fig).

#### Calibrating the ICU bed weighing scale

Calibrating the ICU beds with integrated weighing scales took place during the bed-making process by the care assistants, just before placing the beds in the ICU.

#### Weighing

Throughout the study, patients were weighted twice daily according to the study protocol. The ICU nurses performed the daily weighing and recorded it in the EHR, along with additional materials in the bed. Only loose hanging elements, such as a urinary catheter bag, had to be lifted. This method minimizes the burden on ICU nurses, because one push of a button is sufficient to determine the patient’s weight. Additional materials such as an extra bedsheet (1,1kg), white sheet (0,6kg), pillow (1kg), blanket (1,9kg) or surgical jacket (0,3kg) were also documented in the EHR. To ensure proper implementation of the study weighing method, a multifaceted approach was chosen to inform nurses. Educational materials such as posters, leaflets and e-mails were distributed to the nurses. Furthermore, during the first three weeks of the study, research nurses were physically present in the ICUs during daytime measurements to answer questions.

#### Electronic Health Record

The patient’s weight was recorded in the EHR (EPIC Systems Corporation, Verona, WI). A reminder for weighing the patient was included in the ‘task list’ of the EHR at set time stamps (12:00 PM, 12:00 AM). Weighing procedures were performed according to the study protocol. The nurse documented the patient’s weight and additional materials in the EHR.

#### Updates during the study

Throughout the course of the study, the nursing staff was provided with monthly updates to maintain awareness and interest in the study progress.

### Ethical issues

The study obtained approval from the institutional review board of the University Medical Center Groningen (METc2022/483). It was conducted in accordance with the principles outlined in the Declaration of Helsinki (version 64, October 2013), the European Union General Data Protection Regulation (EU GDPR), the Dutch code of conduct for science practice, and hospital regulations and acts [22, 23].

The institution’s ethical committee waived the formal informed consent requirement under the Human Scientific Research Act [24]. Patients could object to the use of their data for research purposes, which was recorded in the EHR and verified by the dedicated research team.

## Results

### Participants

**Fig 1:**
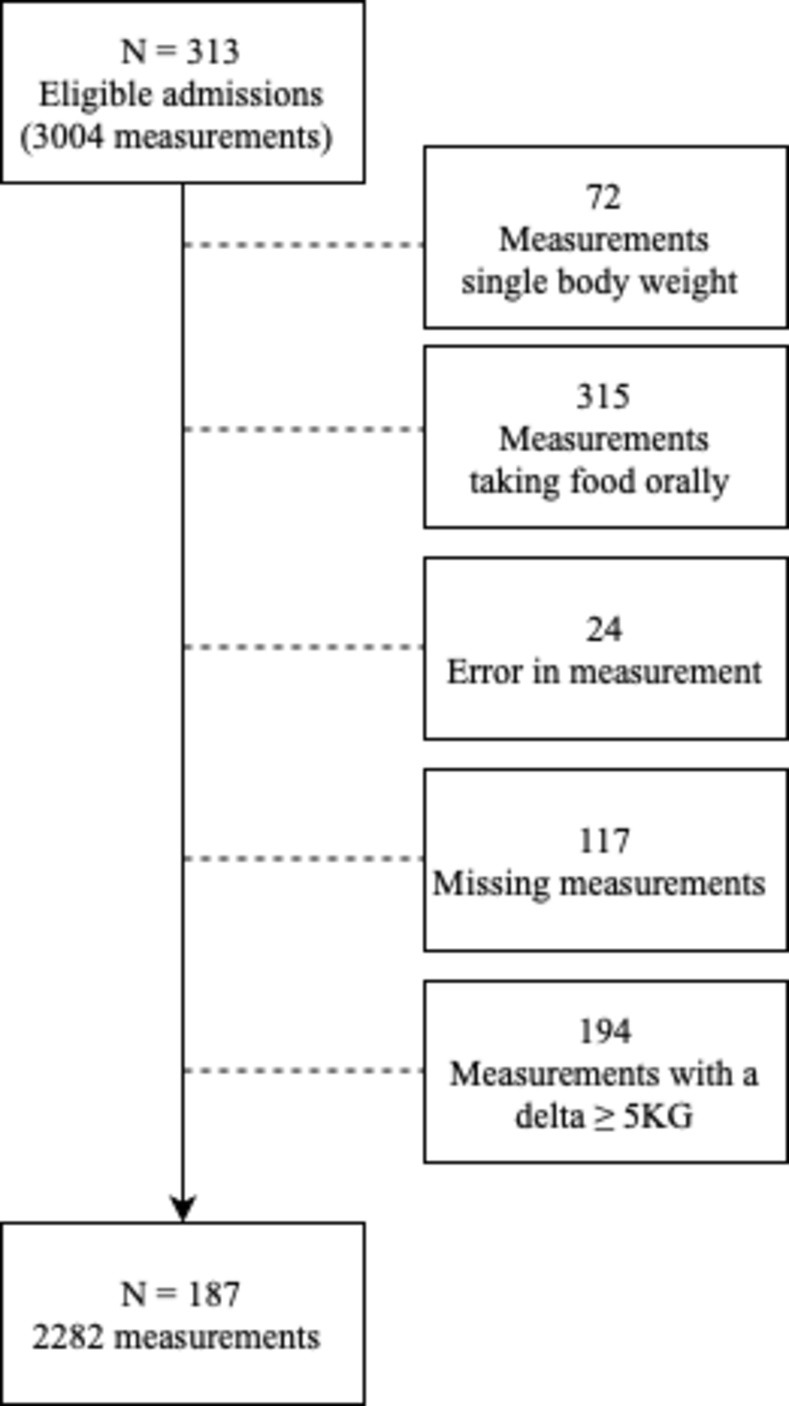
Included measurements.

During the course of the study, a total of 540 patients were admitted to the ICU. Of these patients, 216 were excluded due to a bed without a weighing scale, 11 patients were excluded due to other reasons, such as improperly calibrated beds and death within a short period of time after admission. Therefore, 313 patients met the eligibility criteria. Among this group, a total of 2282 measurements were eligible for inclusion, 722 measurements had to be excluded due to various reasons (Fig 1).

### Demographic data

The mean age of the study participants was 56.4 years (SD, 16.67), and 124 of them were male (66%). The average length of stay in the intensive care unit was 7.8 days, and the majority of patients (115) were admitted for medical reasons such as sepsis and trauma. The mean APACHE VI was 72.8 (SD, 29,3) (Table 1 and S1 Data).

**Table 1:** Patient’s characteristics.

Patients, N	187
Age, mean (SD)	56.4 (16.67)
Sex, Male, N (%)	124 (66%)
ICU LOS (d), mean (SD)	7.8 (7.9)
Reason of admission	
Medical, N (%)	115 (61.4%)
Emergency surgery, N (%)	54 (28.9%)
Planned surgery N (%)	18 (9.6%)
Apache IV score, mean (SD)	72.8* (29.3)

*N = 174, Apache IV score missing for 13 patients due to age (n = 2), transplantation (n = 10), burning wounds (n = 1)

### Findings

#### Primary findings

**Fig 2:**
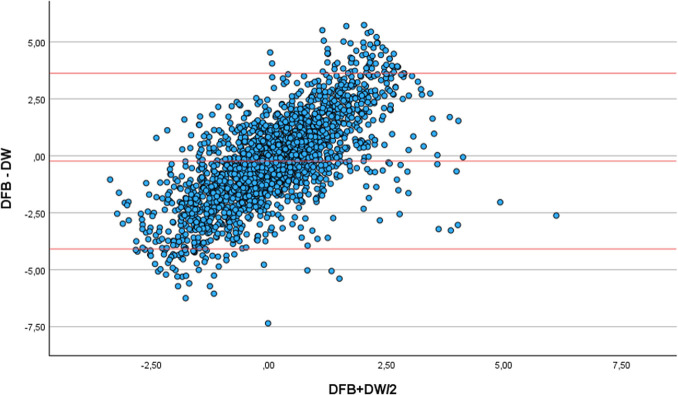
Bland Altman plot for changes in fluid balance and simultaneous body weight. X-axis, mean value of delta fluid balance (DFB) and delta weight (DW). Y-axis, difference between delta fluid balance (DFB) and delta weight (DW).

The measured weights and fluid balances appeared to follow a normal distribution (S2 Data). The Pearson’s correlation between daily changes in weight and fluid balance was weak (r = 0.274, P<0.001). In addition, the Bland Altman plot demonstrated a small mean bias (-0,2304), and the 95% confidence interval (CI) was wide (between 3.63 and -4.09 kg, Fig 2).

An examination of the data comparing weight and fluid balance during the first week and second week of ICU admission revealed a similarly weak correlation in both periods. The Pearson’s correlation for the first week was determined to be 0.330 (P<0.001, and the correlation for the second week was found to be 0.218 (P<0.001).

#### Secondary findings

**Fig 3:**
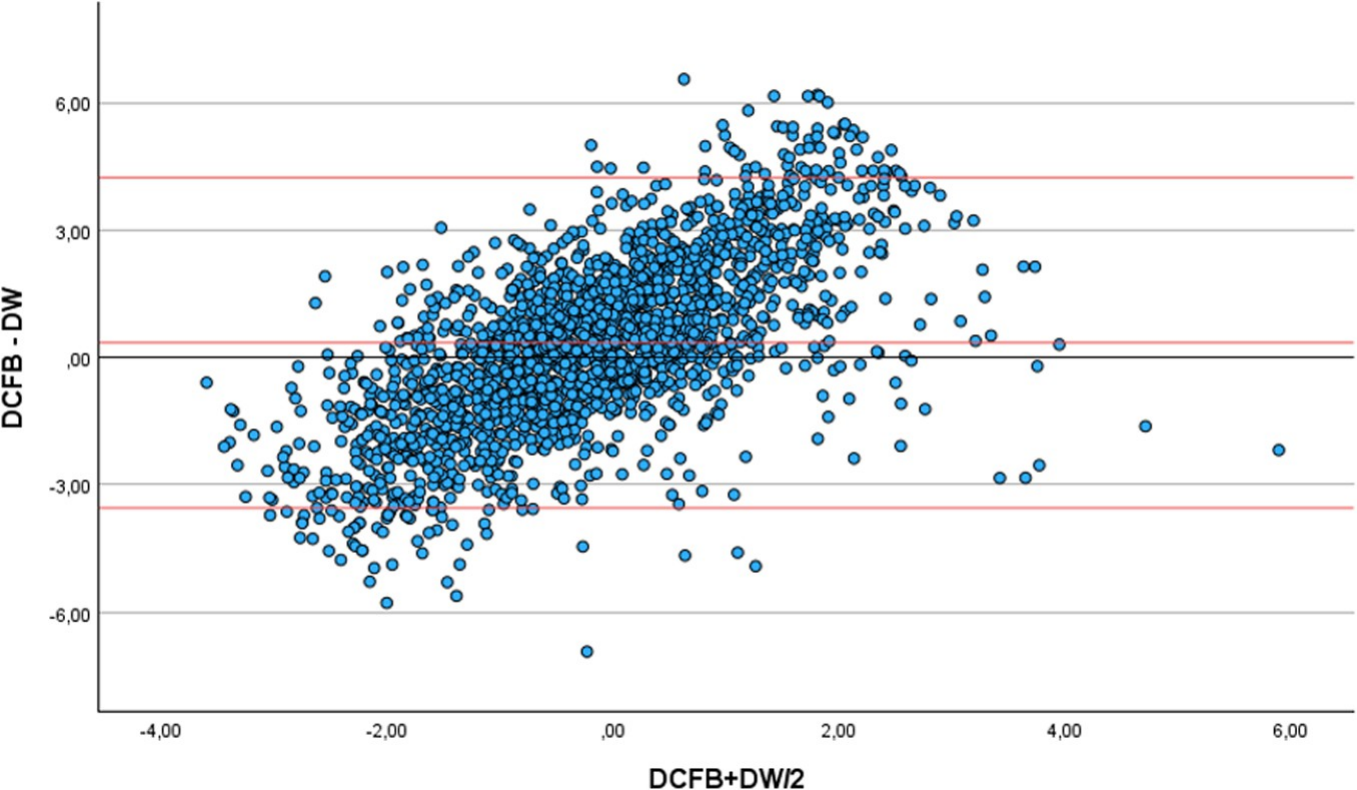
Bland Altman plot for changes in the corrected fluid balance and simultaneous body weight. X-axis, mean value of delta corrected fluid balance (DCFB) and delta weight (DW). Y-axis, difference between delta corrected fluid balance (DCFB) and delta weight (DW).

Examining the corrected fluid balance for IFL, the histogram displayed a normal distribution. Pearson’s correlation was weak 0,268 (P<0.001). The mean difference between measurements was 0,3417 (SD, 1,968), and the corresponding CI was between 4,23 and -3,55kg (Fig 3).

Analysis examining the relationship between weight and the corrected fluid balance for IFL during the initial week of ICU admission and the subsequent week demonstrated a weak correlation in both periods. The calculated Pearson’s correlation coefficient for the first week was 0.218 (P<0.001), while the correlation coefficient for the second week was 0.208 (P<0.001).

## Discussion

### Summary of key findings

We conducted a prospective observational study in which 2282 measurements were performed in 187 patients. Despite the inclusion of a large number of measurements and sufficient statistical power, the correlation between weight and fluid balance was weak (r = 0.274). After adjusting the fluid balance for IFL, the correlation between weight and fluid balance remained weak (r = 0,268). Consistent with the observed correlation, the Bland Altman analysis revealed a wide CI in both the fluid balance and corrected fluid balance versus changes in body weight. In addition, when the data from the first and second week of ICU admission were analyzed separately, the Pearson’s correlation did not change.

### Comparison with previous studies

The findings of our study are inconsistent with the most recent study on body weight and fluid balance changes published in 2021 [2]. Mishra et al., reported a good correlation between weight changes and cumulative fluid balance (r = 0.423, P<0.001). However, it is important to note that only 105 patients were included in the study. In contrast to our study, the patient’s body weight was measured without any additional materials in the bed, and the fluid balance and body weight were performed only once a day. Weighing patients twice a day in bed with additional materials may have introduced measurement errors and potentially explains the weak correlation observed. Additionally, the patient’s gastrointestinal condition may influence the weight. However, the patients were weighted in a clean bed, and no additional materials, such as diapers, were used to collect feces. Similar to our study Schneider et al. found a weak correlation between 12-hour changes in body weight and fluid balance (r = 0,28), even after correction for IFL (r = 0.27) [13]. The group studied was homogeneous (103 patients undergoing cardiac surgery, 414 measurements), and modern technology has improved over the years. Additionally, a study conducted in 2012 investigated the differences in body weight and fluid balance between two consecutive days in ICU patients, also revealing a weak correlation (r = 0,34), even when the fluid balance was corrected for IFL (r = 0,34. They obtained 435 data points in 151 patients [10]. Our study benefits from a larger sample size and heterogonous patients, enhancing the reliability of the results and providing greater measurement accuracy. Though in line with the before mentioned studies, our findings also revealed a rather poor correlation.

Previous studies have demonstrated the poor reliability of automatic fluid balance measurements in the ICU [25, 26]. There are several explanations for this poor reliability, one of them is the complexity and amount of repetition of actions required to accurately track inputs and outputs [5, 7]. Multiple studies indicate that fluid balances are susceptible to false positive and false negative recordings. For example, the inability to measure vomiting or leaking wounds can contribute to inaccuracies in the administration [27]. In addition, the fluid balance is partly generated automatically, and monitoring is limited [5, 7].

### Strengths and limitations

This study has several strengths. A large number of measurements were included in the study, and the percentage of missing data was low (5%). This can be attributed to the study weighing procedure, which was perceived as easy and non-labor-intensive, contributing to a high degree of adherence to the study protocol. In addition, the research team was present at the bedside during the first three weeks of the study to address any questions. During the study period, the research team monitored the completeness of measurements by the ICU nurses on a daily basis. Finally, the inclusion of a diverse patient population with varying demographics in this study allows for generalizability to other ICUs. However, some limitations can be noticed in this study. Weighing was performed without removing additional materials from the bed. Because the measurements were performed individually without control, the additional materials may not have always been documented properly. No assessment of inter-rater reliability was performed because measures were performed individually without cross-checking. In addition, it should be noted that the manufacturer has not tested the bed scale in patients but only in vitro [16]. However, we have performed a small pilot study that confirms that the MultiCare bed is indeed weighing accurately.

### Recommendations for future research

Given the lack of a gold standard for determining changes in volume status, it is imperative to identify the most reliable fluid monitoring approach in the future to define a gold standard. Therefore, separate evaluations of weighing technique and fluid balance assessment should be performed while at the same time focusing on ways to improve the accuracy of both measurements. Furthermore, the use of sensory measurements has the potential to increase the accuracy of both weight measurement and fluid balance assessment, which will hopefully contribute to a gold standard for fluid management over time.

### Implications for clinical practice

This study shows a weak correlation between changes in body weight and fluid balance. Therefore, a combination of the daily fluid balance along with daily weighing should be considered in clinical practice when monitoring fluid management in the ICU. This dual approach can serve as a control mechanism for outliers in measurements of fluid balance or weighing within clinical practice.

## Conclusion

In conclusion, the trends in weight and fluid balance are significant different in ICU patients, even when the fluid balance is corrected for IFL, the correlation between fluid balance and weighing remains weak.

## Supporting information

S1 Fig Scatterplot integrated Multicare ICU bed vs weighing chair. (TIF)

S1 Data Demographic data. (XLSX)

S2 Data Measurement data. (SAV)
