# Supplementary figures and images for: Fluid balance versus weighing: A comparison in ICU patients: A single center observational study
Source: PLoS One. 2024 Apr 26;19(4):e0299474. doi: 10.1371/journal.pone.0299474 (PMC11051658; doi:10.1371/journal.pone.0299474)

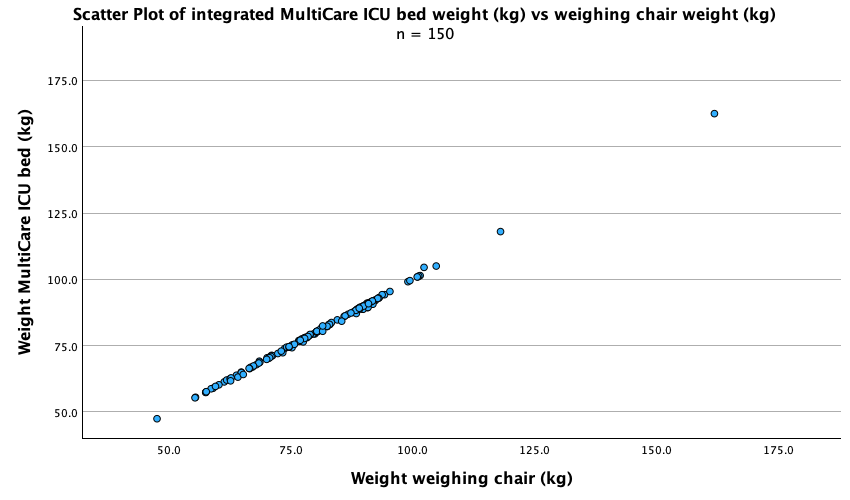

Supplement: S1 Fig — (TIF) [file pone.0299474.s001.tif]
